# Calculation of the Relative Chemical Stabilities of Proteins as a Function of Temperature and Redox Chemistry in a Hot Spring

**DOI:** 10.1371/journal.pone.0022782

**Published:** 2011-08-11

**Authors:** Jeffrey M. Dick, Everett L. Shock

**Affiliations:** 1 School of Earth and Space Exploration, Arizona State University, Tempe, Arizona, United States of America; 2 Department of Chemistry and Biochemistry, Arizona State University, Tempe, Arizona, United States of America; J. Craig Venter Institute, United States of America

## Abstract

Uncovering the chemical and physical links between natural environments and microbial communities is becoming increasingly amenable owing to geochemical observations and metagenomic sequencing. At the hot spring known as Bison Pool in Yellowstone National Park, the cooling of the water in the outflow channel is associated with an increase in oxidation potential estimated from multiple field-based measurements. Representative groups of proteins whose sequences were derived from metagenomic data also exhibit an increase in average oxidation state of carbon in the protein molecules with distance from the hot-spring source. The energetic requirements of reactions to form selected proteins used in the model were computed using amino-acid group additivity for the standard molal thermodynamic properties of the proteins, and the relative chemical stabilities of the proteins were investigated by varying temperature, pH and oxidation state, expressed as activity of dissolved hydrogen. The relative stabilities of the proteins were found to track the locations of the sampling sites when the calculations included a function for hydrogen activity that increases with temperature and is higher, or more reducing, than values consistent with measurements of dissolved oxygen, sulfide and oxidation-reduction potential in the field. These findings imply that spatial patterns in the amino acid compositions of proteins can be linked, through energetics of overall chemical reactions representing the formation of the proteins, to the environmental conditions at this hot spring, even if microbial cells maintain considerably different internal conditions. Further applications of the thermodynamic calculations are possible for other natural microbial ecosystems.

## Introduction

The imprints of distinct geochemical environments can be found in the molecular compositions of microbial genomes and their protein products. For example, transmembrane proteins of ancestral organisms were likely to be depleted in oxygen, paralleling the low oxygen content of Earth's atmosphere in the past [1]. Environmental imprints on proteins can also be found for spatially separated organisms living contemporaneously; the amino acid composition of proteins differ systematically between organisms living at different temperatures [2], [3]. Together with temperature, the chemical properties of the environment are linked to the compositions of gene sequences in hot-spring microbial communities [4], [5].

Although the sequences of proteins must satisfy a complex array of biological requirements, the different biosynthetic costs of amino acids are viewed as one contributing factor to actual patterns of amino acid usage [6], [7]. In some studies, the biosynthetic costs of amino acids have been estimated from metabolic constraints including numbers of phosphate bonds and hydrogen atoms transferred during synthesis from precursors [6], [8]. Those estimates depend on the growth medium and specific metabolic pathways but otherwise do not involve environmental variables such as temperature and oxidation-reduction conditions. Nevertheless, it can be shown that the Gibbs energy change in overall chemical reactions to synthesize amino acids from from inorganic species depends on environmental conditions [9]. The calculations of energetics of overall synthesis reactions can now be done for proteins, where group additivity methods permit assessing standard Gibbs energies of proteins of any amino acid composition [10], [11].

The goal of this study is to use thermodynamic tools to characterize simultaneously the chemical environment and metagenomically derived protein sequences in a hot spring exhibiting large gradients of temperature and oxidation-reduction, or redox, chemistry. The geochemical and biomolecular data are combined using a single model framework based on chemical reactions and their energy changes. The use of a metagenomic dataset in a location where extensive geochemical data are available permits calibration and testing of the model.

One setting where in-depth metagenomic and geochemical information are available is the hot spring known as “Bison Pool”, a flowing, moderately alkaline hot spring in Yellowstone National Park [12], [13]. The water at the source is boiling, and rapidly cools along the outflow channel as a result of exposure to the ambient conditions. Extensive chemical analysis of the water also reveals large gradients of chemical composition such as increase in pH, decrease in sulfide concentration, increase in dissolved oxygen and in oxidation-reduction potential of the water. Prior metagenomic sampling of the microbial communities at five sites from the source to approximately 22 meters down the outflow channel offers a window into the biomolecular composition of these communities. Although the metagenomic sequencing is of the DNA molecules, genes present in the metagenome provide a picture of the proteins that are likely to be used by the organisms.

The first major theme of this study concerns the changes in chemical composition of proteins along the outflow channel. The stoichiometric quantity we investigate is the average oxidation state of carbon in the proteins, which can be calculated directly from the chemical formulas of the proteins. In general, the average oxidation state of carbon in proteins increases down the outflow channel. This effect is present at the level of the whole metagenome and also within different functional classes of proteins. There is a positive correlation between the average oxidation state of carbon in proteins and the oxidation-reduction potential of the surrounding water.

The results of the stoichiometric calculations support a hypothesis that chemical compositions of the proteins reflect processes that tend to minimize the free energy of the system. We applied thermodynamic models to integrate molecular composition with temperature and multiple environmental chemical variables. The second major theme of the paper addresses the relative stabilities of the different classes of model proteins from each sampling site in terms of temperature, pH and oxidation-reduction potential. The major finding of this part of the study is that a redox gradient as a function of temperature traverses the stability fields of the proteins in a way that largely parallels the proteins' spatial distribution. This redox gradient, expressed as activity of dissolved hydrogen, generally parallels estimates derived from measurements of sulfide/sulfate concentrations, oxidation-reduction potential electrodes, and dissolved oxygen, but is more reducing than any of those.

These results help to outline the interrelationships between biomolecular composition and geochemistry in the Bison Pool ecosystem. One use of these models is to quantify gradients in oxidation potential between the water and the interiors of cells and/or biofilms at the temperatures found in the hot spring. Another is to establish the extent to which organisms minimize the energy expenditure involved in formation of biomolecules in specific chemical environments. Generalizing the methods and calculations described below can aid in resolving the effects of chemical gradients, energy minimization and other features of this hot spring and other geobiochemical systems.

## Methods

### Average oxidation state of carbon

The average nominal oxidation state of carbon, 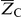, is a quantity related to the different electronegativities of elements involved in the covalent structure of an organic molecule. 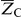 is equal to the sum of the nominal oxidation states of all the carbon atoms in a molecule divided by the number of carbon atoms. The concept of average oxidation state of carbon has found application in various contexts, ranging from balancing organic oxidation-reduction reactions [14] to characterization of organic matter in aerosols [15] and in terrestrial ecosystems [16]. Moreover, a correlation can be observed between the standard molal Gibbs energies of oxidation half-reactions and the average oxidation state of carbon of the organic molecules involved [17]. As with smaller molecules, it is possible to interpret the chemical composition of proteins using the average oxidation state of carbon.

The rules for calculating the formal oxidation states on any carbon atom can be summarized as follows [18]. Each single bond to a more electronegative element (e.g., oxygen, nitrogen, sulfur) contributes 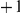 to the oxidation state of a particular carbon atom, while each single bond to a less electronegative element (e.g., hydrogen) contributes 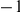 to the oxidation state of a particular carbon atom, and a carbon-carbon bond counts (formally) as zero. Double bonds count doubly. Familiar, though extreme, examples are found with 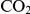 (two double bonds to oxygen; 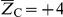) and 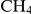 (four single bonds to hydrogen; 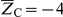). The concept of the average oxidation state can be extended to more complex molecules, for example acetic acid, 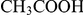, which has 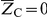. That value is consistent with an oxidation state of 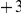 on the first carbon (having three bonds to hydrogen) and 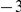 on the second carbon (having one double bond and one single bond to oxygen).

The definition of oxidation state cited in the IUPAC Gold Book [19], [20] states that “… in ions the algebraic sum of the oxidation states of the constituent atoms must be equal to the charge on the ion” (i.e., positive or negative values for cations or anions, or zero for neutral species). We can adopt values for the formal charges of atoms other than carbon, 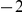 for oxygen, 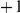 for hydrogen, 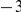 for nitrogen, 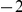 for sulfur, that are consistent with this requirement for amino acids and proteins. The values for nitrogen and sulfur are those that would be assigned to the atoms if they were found in amine groups and sulfide groups, respectively [16]. Writing the formula of glycine as 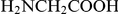, the oxidation state of the first carbon is 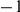 (one bond to nitrogen, two bonds to hydrogen) and that of the second carbon is 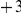 (one double bond and one single bond to oxygen), so the average oxidation state of carbon in the molecule is 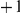. The sum of the formal charges of the atoms, in the order indicated by the formula, is 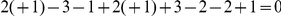, which is equal to the net charge of the molecule.

In many cases, the value of the average oxidation state of carbon is amenable to calculation using only the chemical formula of a molecule, instead of the more tedious accounting for each carbon. Let us use 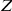 to stand for the total charge on an ion (which becomes zero for a neutral molecule) and let the average oxidation state of carbon be represented by 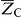. Using formal oxidation states mentioned above for the elements other than carbon, the requirement for algebraic sums of oxidation states of the atoms can be expressed symbolically as
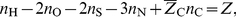
(1)where 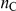, 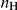, 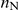, 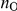 and 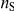 are the numbers of the respective subscripted elements in the chemical formula. Rearranging Eq. (1) gives
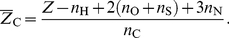
(2)This equation shows that the average oxidation state of carbon in proteins is effectively a linear combination of the elemental ratios H/C, N/C, O/C and S/C.

Note that ionization of the amino acid sidechains in proteins, and other ionization reactions involving only protons, have equal contributions to 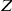 and 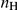 and produce no net effect on the value of 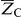. Similarly, polymerization of amino acids, or other reactions involving only the gain or loss of a water molecule, produce no net effect on the value of 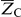 [18]. On the other hand, Eq. (2), and the electronegativity rules outlined above, show that oxidation-reduction reactions in organic compounds are not limited to gain or loss of either hydrogen or oxygen, but that the addition of other heteroatoms (sulfur, nitrogen) to a compound also causes an increase in the overall oxidation state of the molecule [18].

The average oxidation states of carbon in the twenty common amino acids range from 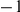 (leucine, isoleucine) to 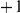 (glycine, aspartic acid, asparagine) and are summarized in Table 1. The values listed for the amino acids in Table 1 span a considerable range but other types of organic molecules are even more or less oxidized [16], [17]. Proteins made up of these amino acids have an average oxidation state of carbon that can be computed as a weighted average of the 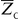 values of the amino acids, or equivalently, using Eq. (2) and the chemical formulas of the proteins. It may be noted that other physical-chemical properties of the amino acids can be correlated with differences in average oxidation state of carbon. For example, four highly hydrophobic amino acids (isoleucine, valine, leucine and phenylalanine) [21] have negative average oxidation states of carbon, which is associated with the high H/C ratios of their sidechain groups.

**Table 1:** Average oxidation states of carbon and number of carbon atoms of the twenty amino acids commonly occurring in proteins.

Amino Acid	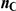	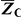	Amino Acid	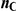	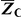
Alanine	3	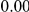	Methionine	5	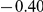
Cysteine	3	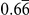	Asparagine	4	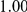
Aspartic Acid	4	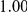	Proline	5	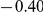
Glutamic Acid	5	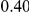	Glutamine	5	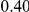
Phenylalanine	9	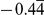	Arginine	6	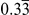
Glycine	2	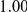	Serine	3	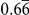
Histidine	6	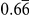	Threonine	4	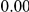
Isoleucine	6	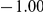	Valine	5	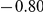
Lysine	6	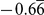	Tryptophan	11	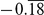
Leucine	6	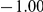	Tyrosine	9	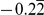

### Relative stabilities of proteins

Chemical thermodynamic methods, borrowed from geochemical modeling applications, can also be used to study the relative stabilities of model proteins from different sampling sites in the hot spring outflow channel. The methods are described conceptually below, followed by description of a specific example.

Four informal definitions help introduce the modeling strategy. 1) *Basis species* are a minimum set of chemical constituents that represent all of the chemical elements and the ionization state of proteins. 2) A *formation reaction* is a chemical reaction to form one mole of a protein from the basis species. The formation reactions of different proteins have different coefficients on basis species because the proteins themselves have different chemical formulas. 3) *Chemical affinity* is energy change during a reaction; positive values mean energy is released, and negative values mean that energy is consumed. A reaction with a higher chemical affinity is more favored to proceed to the product side. 4) *Chemical activity* is related fundamentally to chemical potential and can be thought of as the effective concentration of a basis species or protein.

Let us define one system of interest as a collection of proteins with equal chemical activities interacting with a physical-chemical environment defined by constant values of temperature, pressure, and chemical activities of the basis species. What is the relative stability of one protein compared to another? If the activities of the proteins are equal, the affinities of the formation reactions of the proteins are generally unequal to each other, and the system is not in equilibrium. The most stable protein is identified as the one with the highest chemical affinity of its formation reaction. That is the protein whose formation, at a given chemical activity, releases the most energy, or requires the least energy input.

Now consider the outcome of hypothetical chemical reactions among the proteins, so that different proteins (chemical species) are formed and destroyed at each others' expense, and as a consequence the chemical activities of the proteins change. The temperature and pressure are maintained, and the system is open so that the activities of the basis species are buffered and therefore remain unchanged. One or more specific outcomes of the hypothetical progression of reactions is an assemblage of proteins in a (possibly metastable) equilibrium distribution. In this equilibrium, or minimum-energy state, the chemical affinities of the formation reactions of the proteins are all equal (but might be non-zero), so the hypothetical transformation of one protein to another involves no overall energy change. If the affinities of the formation reactions of the proteins are all equal but less than zero, then the proteins are less stable than the basis species; that system represents a type of metastable equilibrium and a local, not global, energy minimum. Since the system is at equilibrium, the most stable protein is identified as the one with the highest chemical activity – in terms of concentration it has a higher degree of formation compared to the other proteins.

**Figure 1:**
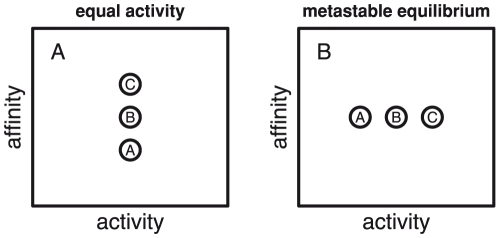
Relative stabilities portrayed in different reference states. In these qualitative diagrams, the same relative stabilities are shown in two different reference states. Species “C” is more stable than “B” is more stable than “A”. Chemical activity is shown on the 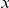-axis, and chemical affinity of formation reaction is shown on the 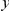-axis. In the equal-activity, or non-equilibrium, reference state (left), the species with the most positive chemical affinity of formation is the most stable. In the equal-affinity, or metastable equilibrium, reference state (right), the species with the most positive chemical activity is the most stable.

The hypothetical systems described above consist of populations of proteins with either equal activities or equal affinities of formation. To a first approximation (under conditions of ideal mixing) the stabilities of the proteins relative to each other are the same in both cases, since the definition of the chemical environment – temperature, pressure and activities of the basis species – is unchanged. Therefore, it is helpful to conceptualize the systems with equal activities of proteins and equal affinities of protein-formation reactions as being different states of a more generic system, defined only by the chemical environment and the identities of the proteins, but not their chemical activities. The relationship between the equal-activity and equal-affinity reference states is shown schematically in Fig. 1. The relative stabilities of species A, B, and C are the same in both panels of the Figure. If an equal-activity reference state is adopted, greater stability goes with higher affinity (Fig. 1a). If the equal-affinity reference state is adopted, greater stability goes with higher activity (Fig. 1b).

In quantifying the relative stabilities of proteins, a choice can be made between the two reference states; either one is valid, but the relative stabilities of the proteins are revealed through different variables. To generate the figures in this paper relative stabilities were quantified using an equal-affinity, or metastable equilibrium reference state. The primary advantage of doing so is the production of equilibrium activity diagrams [22] that are interpreted as depicting the relative stabilities of the proteins in terms of temperature and activities of the basis species. The specific methods used to calculate relative stabilities of proteins starting with an equal-activity reference state, then using a reaction matrix or equilibrium distribution equation to quantify the activities of the proteins at metastable equilibrium are described below.

#### Thermodynamic definitions

An equation for the differential of Gibbs energy (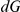) that takes account of reaction progress in a system, formulated by de Donder [23], [24] can be written as
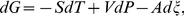
(3)where 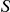, 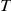, 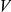 and 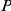 are entropy, temperature, volume and pressure, 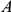 is chemical affinity, and 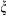 is a reaction progress variable. The chemical affinity of the 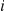
th reaction can be expressed as
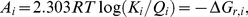
(4)where 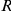 is the gas constant, 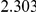 represents the natural logarithm of 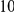, and 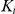 and 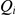 are the equilibrium constant and activity product of the 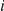
th reaction. The chemical affinity is equal to the negative of the overall Gibbs energy change of the reaction (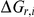). 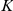 and 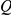 can be calculated from
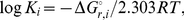
(5)where 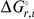 is the standard Gibbs energy of the 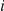
th reaction, and
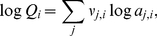
(6)where 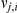 and 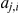 are the reaction coefficient (negative for reactants, positive for products) and activity of the 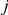
th species in the 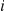
th reaction. In the equations, all operations involving logarithms have a base of 10.

The standard state convention adopted for liquids, including 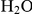, corresponds to unit activity of the pure substance at any temperature and pressure. The standard state convention adopted for aqueous species other than 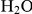 corresponds to unit activity of a hypothetical one molal solution referenced to infinite dilution at any temperature and pressure [25]. The conventional standard molal thermodynamic properties of both the aqueous electron and proton are taken to be zero at all temperatures and pressures [26].

### Calculating relative stabilities of proteins

The case study described below is based on an example described previously [27] for calculating the equilibrium activities of cell-surface glycoproteins (CSG) from *Methanococcus voltae* and *Methanocaldococcus jannaschii*. These methanogenic organisms are not likely to be present in detectable quantities at Bison Pool in Yellowstone National Park, but they nevertheless are common model organisms for studying microbial adaptations to differences in temperature and other environmental characteristics [28], [29]. Clues about the organisms' environments may emerge from comparing the sequence and chemical properties of the two functionally homologous proteins.

The methods described here for calculating the amino acid and chemical composition as well as the standard molal properties of the proteins [11] are currently restricted to only the unfolded peptide molecules and not the carbohydrate constituent of the glycoproteins. In their uncharged states, the peptide chains of these two molecules have formulas of 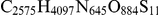 and 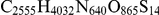, respectively, with sequence lengths of 553 and 530 amino acid residues (UniProtKB accessions Q50833 for CSG_METVO and Q58232 for CSG_METJA; signal peptides were removed). At 25°C and 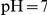, the charges of the ionized proteins calculated using standard Gibbs energies of ionization of the amino acid sidechains and protein terminal groups [11] are 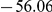 and 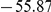. Although the calculation of protein charge, which is based on group additivity, does not take into consideration the effects of interactions between the ionizable groups, it does have the advantage of being sensitive to changes in temperature.

#### Writing formation reactions for residue equivalents

In general, the relative stabilities of proteins of different lengths are of interest. Because of the differences in size, the molal reaction energies can not be directly compared in calculations of relative stability. In many environments, the synthesis of larger molecules, per mole, demands more energy, so for proteins of otherwise equal chemical composition and thermodynamic properties (such as two proteins of different size but with the same relative frequencies of amino acids), the smaller one would generally be thought of as more stable. In more reduced settings, the overall synthesis of organic molecules can actually release energy [30], so the synthesis of larger molecules would be favored. Taking the polymeric nature of the proteins into account, the relative stabilities of proteins of different size can be assessed by first writing formation reactions that are normalized by numbers of amino acid residues. The reactions involve the residue equivalents of the proteins, which have chemical formulas and standard molal properties that are those of the protein divided by the sequence length of the protein. The following two formation reactions are written for the residue equivalents of the two protein homologs:
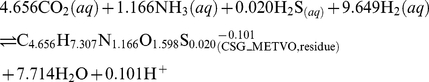
(7)for the protein from *M. voltae* and
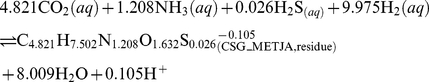
(8)for that from *M. jannaschii*.

The reactions above involve the basis species 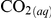, 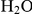, 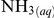, 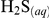, 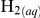 and 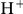, which are the same as used in Ref. [27] except that 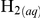 is used here instead of 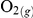. The choice of basis species determines the expression for chemical activities, i.e. the way in which the environmental chemical potentials are quantified. Similarly to components, the set of basis species is valid only if they represent a number of independent variables equal to the dimension of chemical variability in the system. There are unlimited combinations of basis species that would qualify, but the actual choice is usually made to facilitate comparisons with the natural system. For the calculations described in this paper 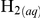 is used instead of 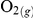 because of the actual formation and metabolic significance of molecular hydrogen in the hot-spring ecosystem [31].

Writing the formation reactions normalized per residue offers insight into the consequences of changing environmental variables on the relative stabilities of the proteins. Because Reactions 7 and 8 are written per residue of the proteins, comparing the reaction coefficients to infer the effect of changing chemical variables on the relative stabilities of the proteins is consistent with an overall reaction between the proteins that is balanced on the protein backbone group. For example, more moles of 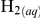 are consumed in Reaction (8) compared to Reaction (7). From specific statements of Eqs. (4) and (6) for both reactions it follows that increasing the activity of 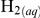 would tend to favor formation of – that is, decrease the energy change of the reaction for – the homolog from *M. jannaschii* more strongly than that from *M. voltae*. The effect of changing activity of hydrogen on the relative stabilities of proteins from *M. jannaschii* and *M. voltae* parallels differences in oxidation state of the natural environments of these two organisms. A likely range of activities of dissolved hydrogen in the mixing zones of submarine hydrothermal vents and ocean water, representative of the environments inhabited by *M. jannaschii*, is 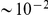 to 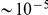 at 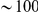
°C [30]. In lower-temperature estuarine sediments, typical of the growth setting of *M. voltae*, lower hydrogen concentrations of 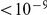 to 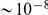 have been observed [32].

Inspection of Reactions 7 and 8 implies that increasing activity of 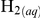 tends to favor formation of the protein from *M. jannaschii* more strongly than that from *M. voltae*. This finding is the opposite of what is implied by the difference between the average oxidation state of carbon in CSG_METJA (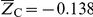) and CSG_METVO (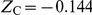); the protein from *M. jannaschii* actually has a higher average oxidation state of carbon. While the average oxidation state of carbon can be derived solely from the chemical composition of the protein, the formation reactions set the stage for understanding relative stabilities of the proteins in terms of reaction stoichiometry, energy, and their relationships to multiple chemical variables represented by the basis species.

#### Calculation of equilibrium constants

The equilibrium constants of each of the reactions can be calculated using the standard Gibbs energies of formation from the elements of the species in the reactions. In this study, the standard molal thermodynamic properties of aqueous species as a function of temperature and pressure were evaluated using the revised Helgeson-Kirkham-Flowers (HKF) equations of state [25], [33], [34], [35]. The equations of state used for liquid 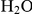 were taken from Refs. [36], [37], [38] as implemented in a Fortran subroutine in the SUPCRT92 software package [39]. Values of the standard molal thermodynamic properties and of the equations of state parameters for the basis species other than 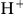 and 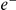 were taken from Refs. [40], [34], [41].

The standard molal thermodynamic properties and equations of state parameters of the proteins can be calculated from amino acid group additivity [11]. In the present study, the CHNOSZ package [27] for the R software environment [42], which includes the group additivity equations for the proteins and the equations of state for calculating standard molal thermodynamic properties as a function of temperature, was used for the calculations. Sample code for performing the calculations for this example is included in Supporting Dataset S1. Combining the sources of data outlined above, values of 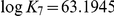 and 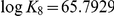 at 25°C and 1 bar can be obtained for these two reactions.

#### Calculation of chemical affinities

The next step is to calculate the chemical affinities of the formation reactions in an equal-activity reference state. Let us use Eq. (4) to write
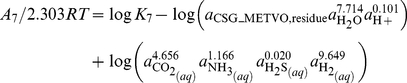
(9)and
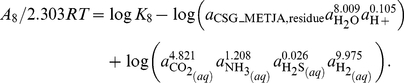
(10)


The activities of the basis species are set to reference values nominally representative of environmental conditions. The activities of the basis species used in this example are taken from Ref. [27]: 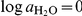, 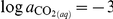, 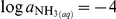, 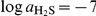, 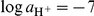 (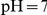) and 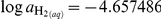. The value for 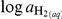 was chosen so that the results would be numerically equivalent to those described in Ref. [27], where 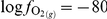 was specified instead. Substituting these values into Eqs. (9) and (10) allows us to write
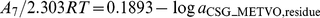
(11)and
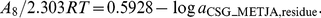
(12)


The activities of the residue equivalents are related to the activities of the proteins as follows. Activity of the 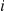
th protein (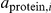) is related to concentration (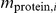, for molality) by
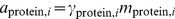
(13)where 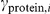 stands for the activity coefficient of the 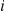
th protein. The total activity of residues in the 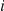
th protein (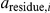) is given by
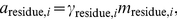
(14)where 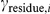 stands for an activity coefficient. The total molality of residues associated with the 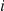
th protein is
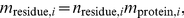
(15)where 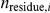 stands for the number of amino acid residues, or sequence length of the 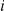
th protein.

Because of the high concentration of metabolites and biomacromolecules in cells, the activity coefficients of proteins in their natural subcellular environments are probably significantly different from unity [43], but available methods for calculating non-ideal behavior of protein solutions are referenced to electrolyte solutions [44] and depend on structural parameters of proteins that would be difficult to deduce from metagenomic sequence fragments. Under these circumstances the activity coefficients of both residues and proteins can be approximated as unity, and Eqs. (13)–(15) can be combined to write
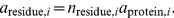
(16)To characterize the affinities in an equal-activity reference state, activities of the proteins nominally given by 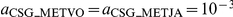 are used for this example. Using Eq. (16), one then obtains reference activities of the residues given by 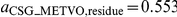 and 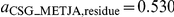 at 25°C and 1 bar.

The reference activities of the residues (computed from equal activities of proteins) can be substituted into Eqs. (11) and (12) to write 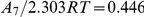 and 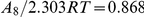. Therefore, on a per-residue basis, the homolog from *M. jannaschii* is more stable under the conditions (temperature, pressure, chemical activities of basis species) stated above. Decreasing the activity of hydrogen below a certain value, or changing the values of one or more variables in a specific manner determined by the reaction stoichiometry and Gibbs energy, would change the outcome so the homolog from *M. voltae* would be the more stable protein.

#### Calculation of the metastable equilibrium activities of proteins: Reaction-matrix approach

Casting the relative stabilities of the proteins into a metastable equilibrium reference state facilitates comparisons on equilibrium activity diagrams. One approach involves a reaction matrix, where a system of equations is constructed based on the formation reactions of the proteins. In metastable equilibrium, the affinities of the formation reactions are all equal. Let us denote this value by 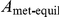. Combining 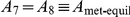 with Eqs. (11) and (12) permits writing
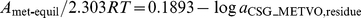
(17)and
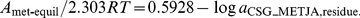
(18)So far this is a system of two equations with three unknowns. A third equation arises from the conservation of activity of residues in the system; recall that activities are additive only if the activity coefficients are unity. Assigning both proteins reference activities of 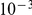, the total activity of residues follows from Eq. (16):
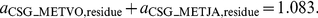
(19)The solution to the system of equations (17)–(19) is 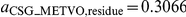, 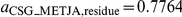 and 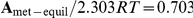. It follows that the metastable equilibrium activities of the proteins (not the residues) are 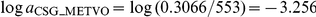 and 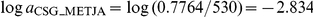. As with the outcome of the equal-activity calculations described previously, CSG_METJA is found to be the more stable protein at the conditions of this example. Changes in temperature, pressure or activities of the basis species would alter these results; in some conditions, for example at more oxidizing conditions specified by lowering the activity of hydrogen, CSG_METVO would instead be the more stable protein.

Each additional protein that is added to the system represents another unknown and another equation like Eq. (17) or (18), so this method is applicable to systems with any number of proteins. Note however that Eqs. (17)–(19), or others that would be written for different systems of proteins, do not constitute a linear system of equations; the unknown activities are summed in the last equation, but the logarithms of activities appear in the former equations. In software, a root finder can be used to solve these equations, leading to slow performance when the relative stabilities of many proteins (hundreds or thousands) are being considered. This performance penalty would not hinder the calculations described in this paper because at most five model proteins for each of the sampling sites are being considered. However, it is useful to consider a different approach, described in the next section, that is computationally more direct and yields identical results.

#### Calculation of the metastable equilibrium activities of proteins: Boltzmann distribution

Let us define, for the per-residue formation reaction of the 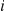
th protein,
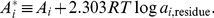
(20)It can be seen by comparison with Eqs. (4) and (6) that 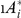 includes all contributions to the chemical affinity of the 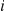
th reaction except for the term associated with the activity of the residue equivalent of the protein of interest. For the per-residue formation reaction of the 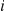
th protein, it follows that
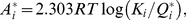
(21)where
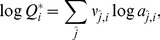
(22)where 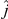 enumerates all of the basis species, but not the protein, in the 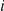
th reaction.

In physical applications, the Boltzmann distribution gives the probabilities of occupation of specific energy levels for systems in thermal equilibrium [45]; analogously for chemical systems it can be used to derive the equilibrium distributions of species [46]. An expression for the Boltzmann distribution, written using the current notation, is
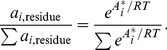
(23)Since the chemical affinity is the negative of the Gibbs energy of reaction, the exponents in Eq. (23) do not carry negative signs, unlike the energy terms in most common representations of the equation [45].

Following the case study above, it can be deduced from Eqs. (11), (12) and (20) that 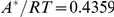, and 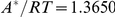. From Eq. (19) it follows that 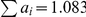. Substituting these values into Eq. (23), one can directly calculate 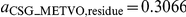 and 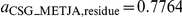. These are the same as the values calculated above using the reaction-matrix approach, and can be combined with Eq. (16) to calculate the metastable equilibrium activities of the proteins. Application of Eq. (23) works as well for systems of three or more proteins and, compared to the reaction-matrix approach, leads to a more efficient implementation in software and faster calculations.

#### Equilibrium activity diagrams

In the example described above, the calculations were carried out at only a single point in temperature-pressure-chemical activity space. The stability calculations can also be performed when one considers the effects of changing temperature, pressure, and/or chemical activities of the basis species, singly or in combination. Interpreting the results of this type of calculation is facilitated by visualizing the relative stabilities of the proteins on equilibrium activity diagrams. In the Results described below, the lines on chemical speciation diagrams show the metastable equilibrium activities of the proteins as a function of a single or composite variable on the 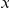-axis. Where two variables are being considered, the fields on predominance diagrams show the protein with the highest metastable equilibrium activity, as a function of the two variables on the 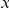
- and 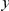-axes.

The thermodynamic calculations and stability diagrams reported below were made using the CHNOSZ software package [27]. The package encodes the equations of state, thermodynamic data, and the group additivity algorithms for proteins cited above. In recent versions of the software, the Boltzmann distribution was implemented for calculating the relative stabilities of proteins, and the calculations reported below use this method. The source code for the calculations reported in this paper, written in the R language [42] and utilizing the functions available in CHNOSZ, is available in the Supporting Information of this paper; Dataset S1 contains code for the example described above, and Dataset S2 contains the code used to produce the figures in the Results.

### Contribution by energy of protein folding to uncertainty in chemical stability calculations

The group additivity algorithm adopted here for calculating the standard molal Gibbs energies of proteins is referenced to unfolded aqueous proteins [11]. Most proteins in their active forms adopt a folded conformation. The energy change for the folding reaction, or change of conformation, is commonly referred to as “protein stability” [47]. The latter nevertheless is distinct from the chemical stabilities being considered in this study, which are based on energies of protein formation. However, the energy change in the folding process contributes some uncertainty, assessed below, to the values adopted here for the standard Gibbs energies of the proteins.

It was estimated that the uncertainty in standard Gibbs energies of proteins inherent in the group additivity algorithm is of the order of five percent [11]. The values of 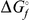 of the non-ionized forms CSG_METVO and CSG_METJA calculated using group additivity are 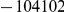 and 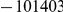 kJ mol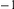 (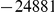 and 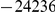 kcal mol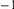), respectively [27]; a nominal 5% uncertainty corresponds to 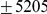 and 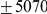 kJ mol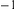 (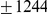 and 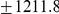 kcal mol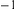). For proteins of comparable size, Gibbs energies of folding of 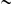
40–80 kJ mol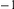 (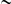
10–20 kcal mol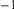) are not uncommon, depending on the temperature [48], [47]. These values are approximately one-one hundredth the magnitude of the estimated uncertainties in the additive standard Gibbs energies of the unfolded proteins. Moreover, the effect of any systematic uncertainty that affects the standard Gibbs energies of the proteins in the same direction (as would the folding process) would tend to cancel in the relative stability calculations. Therefore, not accounting for the energy of protein folding contributes little to the overall uncertainty of the relative stability calculations.

## Results

### Description of field site and metagenomic sampling

**Figure 2:**
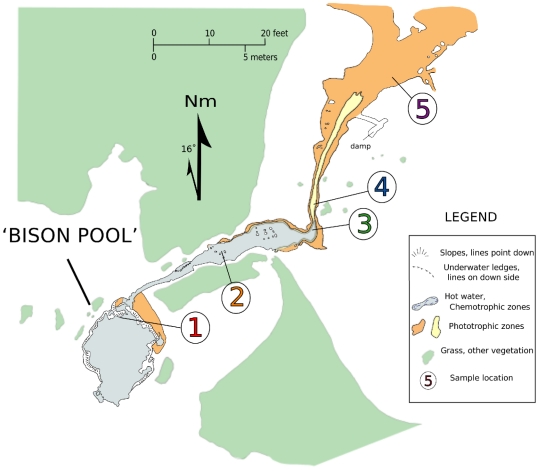
Map of the Bison Pool hot spring system. The map includes locations of the sites where biofilm and geochemical sampling was performed in the summer of 2005.

Chemical and biological sampling was performed in July 2005 at the hot spring known as “Bison Pool” in the Sentinel Meadows in the Lower Geyser Basin of Yellowstone National Park [12]. “Bison Pool” is the unofficial name of a hot spring whose source pool is located at approximately 44.56961°N, 110.86513°W (WGS 84 datum), the closest officially named feature being called Rosette Geyser [49]. A map identifying the sampling sites referred to in this study, based on one found in Ref. [12], is shown in Fig. 2. The spring emits a continuous flow of boiling (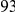
°C), moderately alkaline (pH
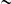
7.5) water, and emerges from within a base of sinter made of silica that has precipitated from the water. The winding outflow channel is occupied by a plethora of biofilms in a striking array of colors. At this and similar springs, the white and pink filaments found at higher temperatures harbor chemotrophic organisms such as *Aquificae* and some Archaea [50], [49]. Yellow, orange and green biofilms (thick mats) found at lower temperatures are predominantly made up of photosynthetic communities of Cyanobacteria and relatives of *Chloroflexi* [51], [13], although archaeal organisms can also be found at the lower temperatures [13].

The available metagenomic and geochemical data were obtained from five sampling sites from the source pool of the hot spring to 22 meters down the outflow channel. Site 3 is notable because it is within the “photosynthetic fringe”, or the transition zone (ecotone) where bright colors indicate the onset of photosynthetic potential [52], [12]. A summary of some of the field- and laboratory-based chemical analyses of the water relevant to this study is given in Table 2. Together with a decrease in temperature down the outflow channel, there is an increase in pH. An increase in the oxidation potential of the water is also apparent from the higher dissolved oxygen and lower sulfide concentrations observed in water sampled away from the source.

**Table 2:** Sampling site identification, distance from source pool, and summary of chemical and molecular sequence data at five sampling sites at Bison Pool^a^.

Site	Code	Distance (m)	T (°C)	pH	Reads^b^	Protein sequences^b^	DO (mg/L)	DIC (ppm)	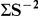 (M)	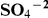 (M)
1	N	0	93.3	7.350	68350	40360	0.173	81.97	4.77E-06	2.10E-04
2	S	6	79.4	7.678	76642	50497	0.776	80.67	2.03E-06	2.03E-04
3	R	11	67.5	7.933	66798	43250	0.9	80.06	3.12E-07	1.98E-04
4	Q	14	65.3	7.995	123327	83790	1.6	78.79	4.68E-07	2.01E-04
5	P	22	57.1	8.257	90921	74082	2.8	78.75	2.18E-07	1.89E-04

a Temperature and pH were measured in the field with hand held temperature/conductivity (YSI, Yellow Springs, Ohio) and pH (WTW, Weilheim, Germany 300i pH meter with SenTix 41 pH electrode) meters. Dissolved inorganic carbon (DIC) was calculated from field titration of alkalinity. Dissolved oxygen (DO) and total sulfide (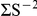) were measured in the field using a portable spectrophotometer (Hach, Loveland, Colorado). Sulfate was measured by ion chromatography in the lab (Dionex, Sunnyvale, California). For additional details see Ref. [55].

b Number of metagenomic reads and number of protein-coding genes available in files downloaded from IMG/M.

The biofilm samples used for metagenomic sequencing were collected at the same time as the water samples used for chemical analysis [12], except for field measurements of oxidation-reduction potential (ORP), which were obtained in 2009. Environmental DNA in the biofilm samples was shotgun-sequenced by the Joint Genome Institute using the Sanger method. The assembly and annotation of protein coding sequences was carried out through an automated pipeline in the Integrated Microbial Genomes with Microbiome Samples (IMG/M) system [53], and sequences used in this study were downloaded from the IMG/M website (http://img.jgi.doe.gov/m).

### Amino acid compositions of model proteins

For this study, FASTA data files containing predicted protein sequences were downloaded from IMG/M using the taxonomic IDs BISONN, BISONS, etc. The letter codes for all the sampling sites are listed in Table 2, together with the total numbers of metagenomic reads and protein-coding sequences for each site.

Because they are derived from shotgun sequencing, most of the inferred protein-coding sequences are actually fragments of whole genes. It would be possible to select specific types of homologs, align the sequence fragments, and use the aligned positions in the stoichiometric and thermodynamic calculations described below. However, the calculation of the average oxidation states of carbon requires only the chemical formula of the proteins, and the calculation of the standard Gibbs energies of the proteins as described in the Methods only requires the amino acid compositions of the proteins. Therefore, in this study, model amino acid compositions were used to represent averages of groups of protein sequences in the metagenome.

The five “overall model proteins” have average amino acid compositions that were calculated as the average of all inferred protein sequences, including fragments, identified in the metagenome at each site. The amino acid compositions of the overall model proteins were calculated by summing the amino acid counts of all sequences at each site and dividing by the total number of sequences at each site. Accordingly, the model proteins are not whole proteins, but instead have fractional amino acid frequencies. The amino acid compositions are listed in Supporting Dataset S3.

The average amino acid compositions were also calculated for “classified model proteins” in twenty functional classes each corresponding to a keyword in the sequence annotations reported in IMG/M. The keywords were selected based on their frequencies in the annotations and represent a variety of functions and cellular structures, but are neither comprehensive nor mutually exclusive. The keywords and number of identified sequences are listed in Table 3. The classification with the highest number of inferred protein sequences is “transferase”, with a total of 15768 sequences across all five sampling sites, or ca. 5.4% of all of the protein sequences in the metagenome. The classification with the fewest number of sequences is “phosphatase”, with a total of 2260 sequences, or about 0.8% of the metagenome. All the keyword searches were case-insensitive and any match was accepted (e.g., an annotation including the word “transporter” was matched by the “transport” keyword), except for “reductase”, which was only matched to the beginning of a word in the annotation (e.g., annotations including the word “oxidoreductase” were not matched by the “reductase” keyword).

**Table 3:** Annotation terms, total number of sequences used to construct the classified model proteins, and 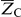 of the classified model proteins (for site 1 only; entries are ordered by decreasing 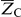).

Classification	Sequences	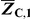	Classification	Sequences	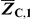
hydrolase	4326	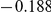	kinase	6123	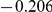
transcription	3747	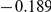	signal	2377	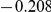
reductase	2905	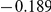	ATPase	7983	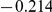
dehydrogenase	9567	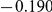	transferase	15768	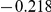
synthetase	5979	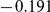	ribosomal	3598	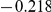
phosphatase	2260	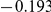	protease	2929	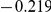
peptidase	3635	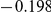	oxidoreductase	3803	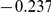
synthase	8561	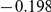	transport	11029	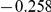
periplasmic	2784	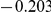	membrane	5194	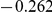
transposase	2845	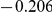	permease	4886	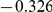

### Average oxidation state of carbon of model proteins

**Figure 3:**
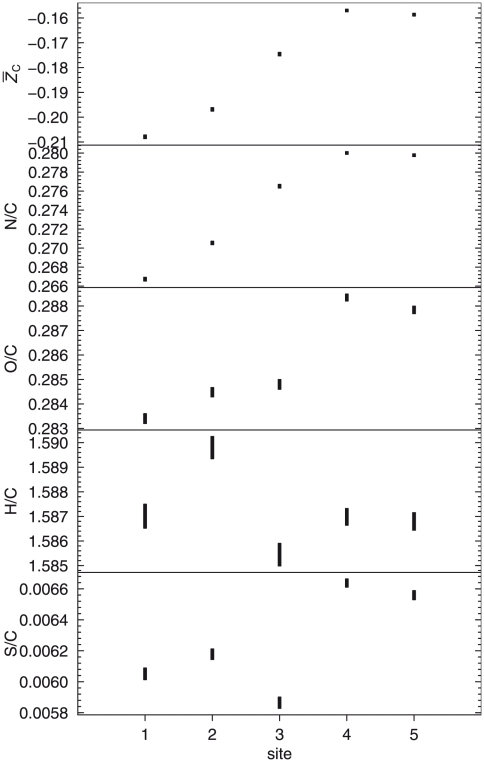
Elemental ratios and average oxidation state of carbon in protein sequences. Elemental ratios were calculated from the chemical formulas of all protein sequences available in the Bison Pool environmental genome, and average oxidation state of carbon (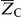) was calculated using Eq. (2). The bars are centered on the means, and the heights of the bars represent the 95% confidence intervals derived from a bootstrap analysis.

To characterize the changes in the compositions of proteins across the sampling sites, we first calculated the elemental ratios and average oxidation number of carbon (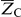) of all the protein sequences available in the metagenome for each sampling site. The 95% confidence intervals around the mean values were calculated from a bootstrap analysis (nonparametric, ordinary bootstrap, 1000 replicates) performed using the “boot” package for the R software environment [42]. The results are plotted in Fig. 3 and the numerical values given in Supporting Dataset S4.

The S/C, O/C and N/C ratios shown in Fig. 3 exhibit an overall increase with distance from the hot-spring source, but there is a decrease in S/C and O/C of the proteins from site 3. The H/C ratio rises sharply between sites 1 and 2 and then decreases, with the proteins at site 3 again having a relatively lower value. The combined effect of the elemental ratios accounts for the trend in 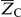 appearing in Fig. 3, which can be described as increasing with distance from the hot-spring source. The chemical compositions of the proteins at sites 4 and 5 are more similar to each other than to the other sites, and the overall trends for elemental ratios and 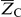 show a slight reversal at these two sites.

**Figure 4:**
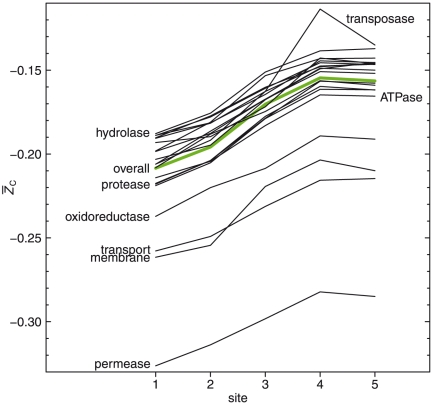
Average oxidation state of carbon in model proteins along the outflow channel. The values of 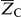 calculated using Eq. (2) for the overall model proteins for each sampling site are shown by the bold green line, and those for each of the 20 classes of model proteins listed in Table 3 are shown by the thin lines. For clarity, only selected classes are labeled; hydrolase and protease are the ones with highest and lowest values of 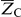 among the main group of lines at the top.

The average oxidation state of carbon of overall and classified model proteins is shown in Fig. 4a. Because of the number of lines plotted in this figure only selected ones are labeled. The others can be identified by referring to the values of 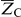 for the model proteins for site 1 listed in Table 3. Whatever the classification of the model protein, there in an increase in 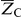 going from site 1 to site 5, and in most cases an increase between each of sites 1 through 4. Generally, there is a slight decrease in the value of 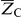 between sites 4 and 5. The differences between the different classes of model proteins are profound: the oxidoreductases, transport and membrane proteins, and especially permeases, all have lower oxidation states of carbon than the others. This result is not surprising, given the greater abundance of hydrophobic sidechains in these predominantly membrane-associated [54] proteins. The model proteins that have the highest oxidation states of carbon are hydrolase at sites 1–3 and transposase at sites 4 and 5. That the transposase model proteins at sites 4 and 5 are more oxidized than other model proteins is noteworthy because at site 1 the transposase model protein has a value of 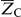 that is only a little greater than that of the overall model protein.

Some relationships can be observed between the chemical composition of proteins and the chemical characteristics of the water. On the whole, there is a positive correlation between the values of 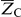 of the model proteins and the field measurements of dissolved oxygen, and a negative correlation with total sulfide concentrations listed in Table 2. The correlations suggest that analysis of the energetics of the protein formation reactions could be used to combine protein composition and hot-spring chemistry in a thermodynamic model. In the following sections results are presented from a thermodynamic analysis that describes the relative stabilities of proteins in terms of temperature, pH, oxidation potential and other environmental variables.

### Relative stabilities of model proteins: Metastable equilibrium

Residue-normalized formation reactions for the overall model proteins are listed in Table 4. These reactions are written in terms of the basis species 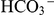, 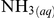, 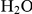, 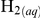, 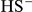 and 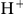, which correspond to inorganic sources of the major elements in the proteins. Because the number of amino acid residues in each of the model proteins is the average of the lengths of metagenomically derived protein sequences, including many fragments, the number of amino acids in each of the model proteins in Table 4 does not necessarily reflect the actual lengths of protein sequences in the microbial organisms in the hot spring.

**Table 4:** Numbers of amino acid residues, average oxidation state of carbon, and per-residue formation reactions normalized per residue for overall model proteins for different sampling sites.^a^

Site	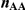	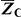	Reaction
1	199.13	−0.208	5.125 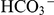 +1.357 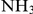 +0.029 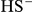 +10.784 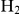 +5.154 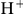 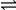 13.924 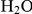 + 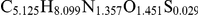
2	184.35	−0.196	5.076 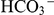 +1.366 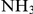 +0.029 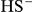 +10.649 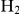 +5.105 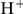 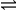 13.788 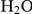 + 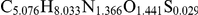
3	195.48	−0.171	5.023 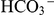 +1.388 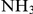 +0.027 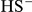 +10.473 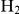 +5.050 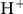 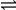 13.639 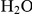 + 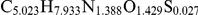
4	191.80	−0.154	4.966 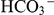 +1.389 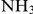 +0.030 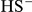 +10.315 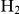 +4.996 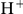 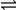 13.467 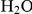 + 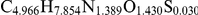
5	189.40	−0.156	4.972 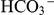 +1.389 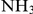 +0.030 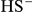 +10.333 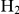 +5.002 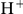 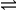 13.487 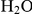 + 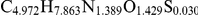

a The amino acid compositions of the model proteins are the bulk averages of all metagenomically derived protein sequences at each sampling site. The values of 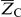 (average oxidation number of carbon) were calculated using Eq. (2).

Because the reactions in Table 4 are written for the formation of the residue equivalents of the model proteins, the reactions are effectively balanced with respect to the protein backbone group. Consequently, the stoichiometric reaction coefficients are independent of the sizes of the model proteins and can be compared with each other in a first approximation to assess the effects of changing chemical conditions on the relative stabilities of the model proteins. For example, the coefficients on 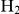, appearing on the reactant side of the reactions, decrease in order of increasing distance from the hot spring source, except for the last two sites, where the pattern is reversed. Therefore, increasing the chemical activity of 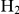 tends to decrease the energy demand of forming overall model proteins at the high-temperature sites more strongly than the others.

The intensive variables used in the equilibrium calculations are temperature (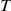), pressure (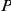), and the chemical activities of the basis species and proteins. Activity coefficients of all species can be set to unity, so, for aqueous species, chemical activities were taken to be equivalent to concentrations in molal units. As noted above, the activity coefficients of proteins in subcellular conditions are currently not amenable to general calculation. In addition, the concentrations of major ions in the water [55] are low enough that setting the activity coefficients of the basis species to unity is a tolerable first approximation. In the calculations reported below, the variables held constant were 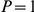 bar, 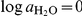, 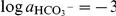, 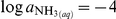, 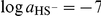. The activities chosen for 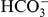 and 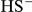 are based on the measurements of dissolved inorganic carbon and sulfide listed in Table 2, while that of 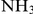 is a nominal value. The calculations of metastable equilibrium were referenced to unit total activity of the amino acid residues in the system. The other variables (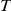, pH and 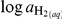) were used as exploratory variables as described below.

**Figure 5:**
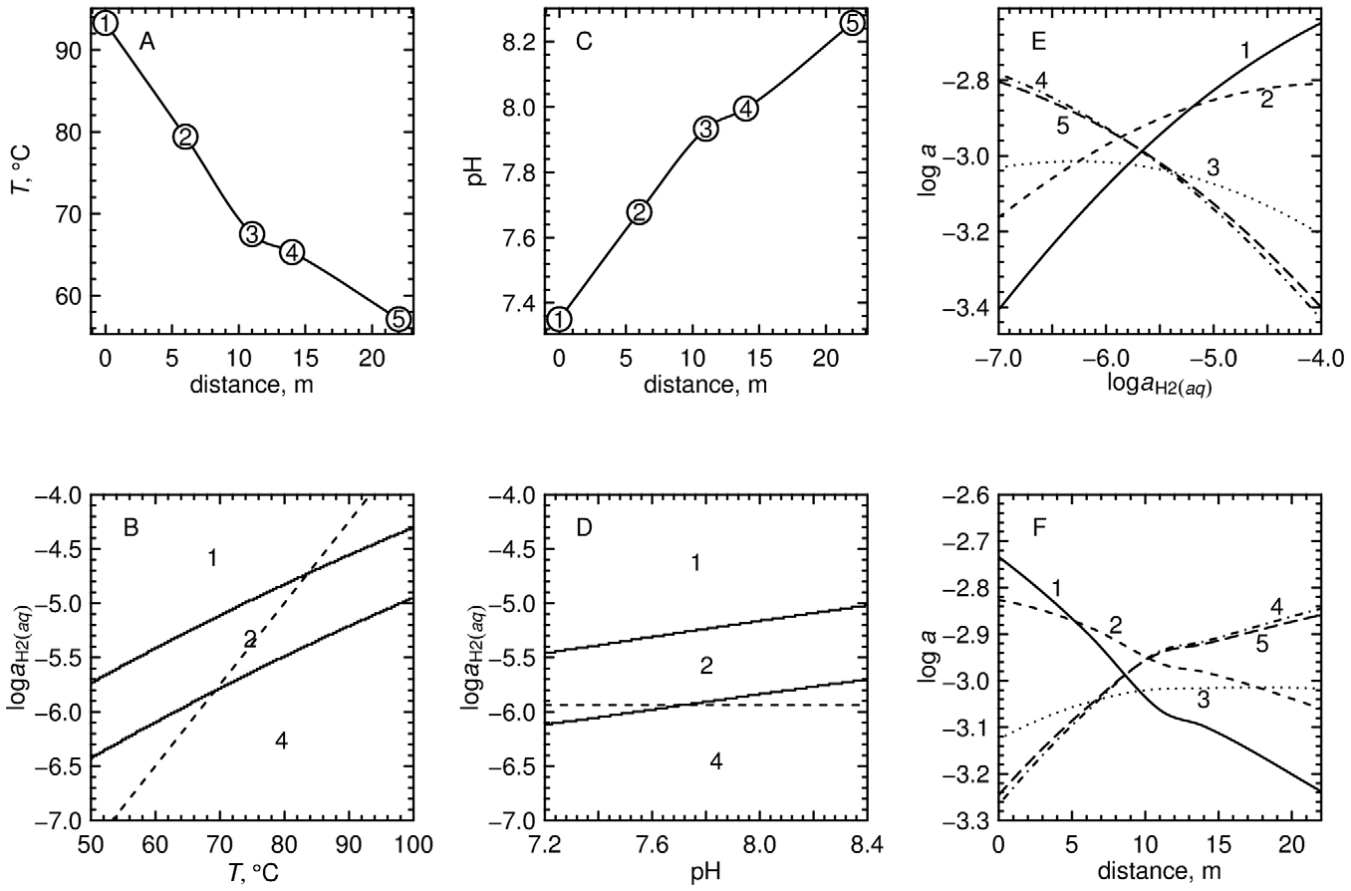
Analysis of relative stabilities of overall model proteins for sampling sites. Panel (*a*) is a plot of the measured temperatures as a function of distance with a smooth curve connecting the points. Panel (*b*) is a plot of the relative stabilities of the overall model proteins as a function of 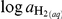 and temperature at the pH of site 3 (7.933). Panel (*c*) is a plot of the measured pHs as a function of distance with a smooth curve connecting the points. Panel (*d*) is a plot of the relative stabilities of the overall model proteins as a function of 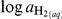 and pH at the temperature of site 3 (67.5°C). The dashed lines in (*b*) and (*d*) depict Eq. (24). Panel (*e*) is a plot of the relative stabilities of the overall model proteins as a function of 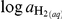 at the temperature and pH of site 3. Panel (*f*) is a plot of the relative stabilities of the overall proteins as a function of the combination of changes with distance of measured temperature and pH and modeled 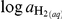 (using Eq. 24).

The results of computations of relative stabilities of the overall model proteins are depicted in Fig. 5. Values of temperature measured at each site in the hot spring (Table 2) are shown in Fig. 5a. Stability calculations were performed for a system composed of the five overall model proteins, using the pH measured at site 3. The most stable overall model proteins as a function of temperature and 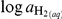 are shown in the equilibrium predominance diagram in Fig. 5b. The temperature range in this diagram is somewhat larger than the measured range of temperature in the hot spring, and the range of 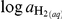 was set to encompass the stability fields of the proteins.

Values of pH measured at each site are shown in Fig. 5c. Stability calculations using the temperature measured at site 3 lead to the equilibrium predominance diagram shown in Fig. 5d. The pH range in this diagram is slightly larger than the measured range of pH in the hot spring.

Note that in Figs. 5b and d, only the overall model proteins from sites 1, 2 and 4 appear, going from high to low values of 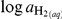 in that order. Increasing pH at constant 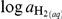 and temperature (Fig. 5d) moves toward the relative stability fields for the overall model proteins that are more distal from the hot-spring source; this pattern is congruent with the pH differences between sampling sites in the hot spring. On the other hand, decreasing temperature at constant 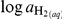 and pH (Fig. 5b) moves toward the relative stability fields for the overall model proteins that are more proximal to the hot-spring source; this pattern is incongruent with the temperature gradient in the outflow channel of the hot spring.

If it were representative of the chemical gradients in the hot spring, the thermodynamic model used here would generate a pattern of relative stabilities of the model proteins that reflects their geographical distribution. It is apparent from the above findings that this is not possible if 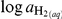 is constant along the outflow channel. Instead, the dashed lines in Figs. 5b and d and the equilibrium chemical activities of the proteins shown in Figs. 5e and f are consistent with changing 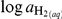 in the model together with the measured changes in temperature and pH and 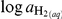, and were derived by considering the relative stabilities of many classes of model proteins as described below.

### Operational equation for activity of hydrogen

**Figure 6:**
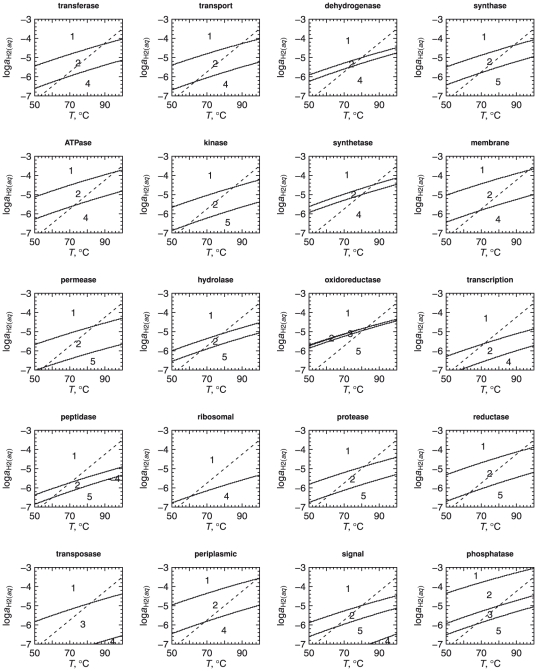
Equilibrium predominance diagrams for classified model proteins. For each class of model proteins, the fields in these predominance diagram represent the model protein with the most positive equilibrium activity as a function of 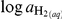 and temperature. pH was set to the value listed in Table 2 for site 3 and the chemical activities of the other basis species were set to reference values specified in the Results section. The dashed line in each diagram indicates values of 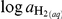 calculated using Eq. (24).

Figure 6 contains 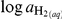-temperature equilibrium predominance diagrams for the 20 classes of model proteins listed in Table 3. These figures were constructed in a manner analogous to Fig. 5b. For each class of model proteins shown in Fig. 6 the protein from site 1 is relatively stable at higher values of 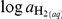, and the protein from site 4 or 5 occupies the low-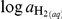 portion of the diagram. Other details differ between the various classes of model proteins; for example, the model protein for phosphatase at site 1 is stable relative to the model proteins for phosphatase at other sites only at highly reduced conditions. Compared to other classes of proteins, the predominance field for the model protein for oxidoreductase at site 2 is much smaller, and that for the ribosomal proteins at site 2 does not even appear. In spite of these differences, the overall resemblance of the plots in Fig. 6 to each other and to Fig. 5b indicates that different subsets of the metagenome have similar relative stability relationships.

The dashed lines in Fig. 5b and d and Fig. 6 denote values of 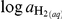 that are given by the function
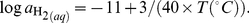
(24)This equation is the result of a graphical regression of the plots in Fig. 5b and Fig. 6 such that the values of 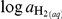 as a function of temperature give a progression in the stability fields of a majority of the model proteins that is similar to the geographical distribution of the proteins. For example, in Fig. 5b, the line given by Eq. (24) encounters the stability fields for the overall model proteins for sites 1, 2 and 4, in that order, with decreasing temperature.

Note that the plot in Fig. 5d is drawn for a single temperature (that for site 3 listed in Table 2), so the line for 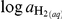 is horizontal in this figure. The line crosses the predominance field boundary between sites 2 and 4 at pH 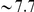, which is close to the measured pH of site 3 (Table 2). The absence of a predominance field for the model protein for site 3 in this figure and in most plots shown in Fig. 6 suggests that generally the proteins at site 3 are less stable than the model proteins for other sites, even under the specific conditions of site 3.

### Effects of other variables

The model developed here incorporates spatial gradients of pH and activity of hydrogen, but in principle other chemical variables could also contribute significantly to the relative stabilities of the proteins. For example, the concentration of dissolved sulfide decreases by more than an order of magnitude between the source of the hot spring and the most distal sampling site (Table 2). However, the consequences of that gradient on the relative stabilities of any two proteins is proportional to the difference in the reaction coefficient of sulfide between the two reactions. In Table 4, the largest difference between reaction coefficients on 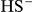 is only 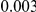, which is much smaller than the differences in the relative stabilities of the proteins (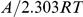 for any of the sites listed the Table). Accordingly, the sulfide concentration in the model was set to a constant since the changes seen in the hot spring have a smaller effect on the relative stabilities of the proteins compared to temperature, pH and 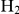. For comparison, the difference between reaction coefficients on 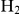 in the formation reactions of overall model proteins 1 and 2 is 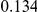, while the difference in relative stabilities of the proteins is 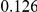, so a one-order-of-magnitude decrease in the activity of 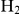, by itself, is enough to increase the relative stability of the model protein for site 2 over site 1.

Conversely, although the coefficients on 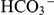 in the reactions shown in Table 4 differ from each other more than those on 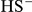, the measured concentrations of dissolved inorganic carbon differ by not more than 4 ppm between the sites (Table 2). Therefore, the small changes in the corresponding activities of 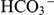 would not be expected to significantly alter the chemical affinities of the reactions relative to each other, so, as with sulfide, the activity of 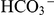 was set to a constant value.

The calculations described above used a nominal value of 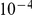 for the activity of 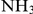. Total ammonia was not detected above 0.01 mg/L (0.6 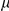
mol) in spectrophotometric analysis of water at the hot spring [55], so it is reasonable to ask whether lowering the activity 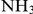 to 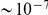 has a major effect on the calculations of relative stabilities. The interdependence of equilibrium activities of the basis species can be assessed by writing a reaction representing the transformation between two proteins, for example the overall model proteins for sites 2 and 4. Adding reaction 4 in Table 4 to the opposite of reaction 2 yields the following reaction:
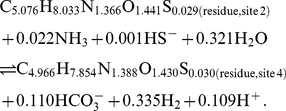
(25)It follows from Eqs. (4)–(6) that, if the chemical affinity of Reaction 25 and the activities of all other reactants and products remain unchanged, decreasing 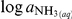 by 3 results in a decrease in the calculated 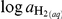 of 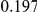 (i.e., 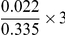). Likewise, reconnaissance calculations indicate that decreasing the activity of 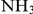 to 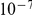 generally results in a lowering of the equal-activity lines shown in Fig. 6, with a more pronounced effect for the lower equal-activity lines, which in some cases shift downwards by at about half a 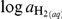 unit. Therefore, refinement of the calculations described here may yield results that support modifying Eq. (24) to have a somewhat steeper slope and lower intercept. Nevertheless, without any direct measurements of the total ammonia concentration, such refinements remain speculative.

### Relative stabilities of model proteins: Chemical affinities

Chemical affinities for the per-residue formation reactions of the overall model proteins in an equal-activity reference state calculated for sites 1 to 5 are listed in Table 5. The calculations used values of temperature and pH listed in Table 2, 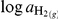 for each of these sites taken from Eq. 24, and activities of the other basis species given above. The activities of the residue equivalents of each of the model proteins were set to unity. All of the reactions listed in Table 4 are endergonic reactions, which is apparent from the negative values of chemical affinity that are shown in Table 5. However, the chemical affinities are less negative at the higher-temperature, more reduced conditions, which is consistent with a previous comparison of the energetics of biomass synthesis under oxic and anoxic conditions [56].

**Table 5:** Chemical affinities for the reactions in Table 4 at the model conditions for each site.^a^

Reaction	Site 1	Site 2	Site 3	Site 4	Site 5
1	**−18.720**	**−27.894**	−35.386	−36.822	−42.265
2	−18.846	−27.914	−35.319	−36.740	−42.120
3	−19.120	−28.053	−35.349	−36.749	−42.051
4	−19.270	−28.080	**−35.276**	**−36.657**	**−41.888**
5	−19.254	−28.078	−35.285	−36.668	−41.907

a The chemical affinities of the reactions are in dimensionless values (i.e., 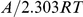) calculated using the temperature and pH of the sampling sites listed in Table 2, 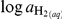 from Eq. (24), chemical activities of the other basis species described in the Results, and chemical activities of the proteins equal to unity. The charges and Gibbs energies of ionized proteins (not shown in Table 4) calculated using group additivity were considered in the calculations of chemical affinity. Bold entries in each columns indicate the reaction with the highest calculated chemical affinity.

Examination of Table 5 shows that the reaction with the greatest chemical affinity at site 1 is that for the overall model protein for that site. In contrast, at the conditions of sites 3–5 the formation, per residue, of the model protein for site 4 is the least energetically demanding. The affinities listed in Table 5 are calculated for the formation reactions of the proteins normalized per residue, but comparison of the relative stabilities of the proteins (as is done on the predominance diagrams) requires further accounting for the relative lengths of the model proteins. Equation (16) was used to account for different lengths when constructing equilibrium activity diagrams for proteins. Analogously, by subtracting the logarithm (base 10) of the number of amino acids present in each of the model proteins from the values listed in Table 4, one obtains length-corrected affinities per residue that can be compared with the equilibrium activity diagrams. For example, performing this operation on the second column of Table 5 shows that the model protein from site 2 is the most stable, even though the per-residue reaction for site 1 has a greater chemical affinity, before applying the length correction. The outcome is in accord with the progression, from sites 1 to 2 to 4, in the relative stabilities of the overall model proteins apparent in Figs. 5b and d.

### Combined analysis of temperature, pH and oxidation potential

Returning to the metastable equilibrium (equal-affinity) reference state, the equilibrium activities of the proteins are plotted in Fig. 5e as functions of 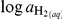 at constant temperature and pH (corresponding to site 3) for a total activity of residues equal to one. Nowhere does the equilibrium chemical activity of the overall model protein for site 3 rise above all the others, which is consistent with Fig. 5b, but it is also apparent that the activity for this model protein maximizes at intermediate values of 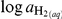. The relative instability of the model proteins for site 3 throughout the different classes of model proteins is apparent from the low frequency of predominance fields representing this site appearing in Fig. 6. It can also be seen in Fig. 5e that the overall model proteins representing sites 4 and 5 are similar to each other in terms of their relative stabilities.

To portray the effects of changing temperature, pH and 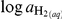 simultaneously, all of these variables are projected along the 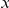-axis (“distance”) in Fig. 5f. The values of temperature and pH at any point along the distance axis are taken from the curves shown in Figs. 5a and c, and the values of 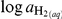 are calculated using Eq. (24). Fig. 5f has the advantage that the relative stabilities of the model proteins are shown as a function of a spatial variable and can therefore be compared with the physical location of the sampling sites in the hot spring.

**Figure 7:**
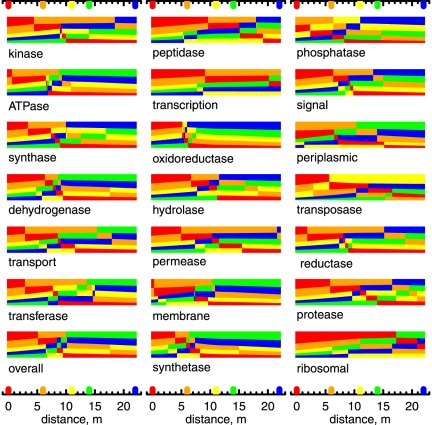
Equilibrium degrees of formation of model proteins in 21 classes. The locations of the sampling sites are indicated by the colored tick marks. The degrees of formation of the five model proteins in each class are shown by the heights of the color-coded bars. At any point on the distance axis, the bars are stacked in order of relative stability, so that the most stable (highest equilibrium degree of formation) is at the top.

In order to visualize the relative stabilities of all of the groups of model proteins on a single figure, the equilibrium activities (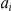) of the proteins were transformed into equilibrium degrees of formation. The degree of formation of the 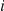
th model protein (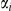) is given by
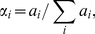
(26)where the summation occurs over all of the model proteins in the calculation, which in this case is five (one for each sampling site). Since 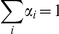, the degree of formation of any protein can be visualized as a fraction of a bar of unit length. The equilibrium degrees of formation of the overall and classified model proteins are shown as a function of distance in Fig. 7. In this figure, the color code refers to the five sampling sites, and the height of the bars represents the equilibrium degree of formation of the indicated model protein. At any point along the distance axis, the bars are stacked with the most relatively stable model protein on the top. The locations and color codes of the sampling sites are indicated by the tick marks.

Fig. 7 permits a visual test of the overall goodness of fit of Eq. (24) to the geographical relationships of the sampling sites and also helps in identifying outliers. At the high-temperature end, the most stable model protein is usually that from site 1, and rarely from site 2 (phosphatase, periplasmic). At the low-temperature end, the most stable protein is usually that from site 4 or 5 (with approximately equal frequency) and only occasionally from sites 2 (transcription) or 3 (transposase). The only model proteins for site 3 that are the most relatively stable over any part of the combined chemical gradient are those for transposase (over the mid- to low-temperature range), oxidoreductase and phosphatase (only at moderate temperatures). The transition zone occurring toward the middle of the plots is sometimes associated with an increase in the relative stability of the model proteins for site 3 (e.g., transferase, synthase), but in many other cases the equilibrium degree of formation of the model proteins for site 3 minimizes relative to the other sites. The overall relative instability of the proteins from site 3 is can be attributed to constraints on their amino acid compositions that also account for the shifts in O/C, H/C, and S/C for these proteins from the overall trends between sites apparent in Fig. 3. A major exception is transposase, for which the model proteins from site 3 are relatively stable over much of the chemical gradient. As in the general case, the relative stabilities are conditioned by trends in amino acid composition that also affect elemental ratios, apparent in Fig. 4 as a high oxidation state of carbon of the transposase model proteins at sites 4 and 5.

### Measurements of oxidation-reduction potential

Eq. (24) represents a proposal for the temperature dependence of the activity of hydrogen derived from the relative stabilities of the model proteins. It can be compared with a variety of other measurements that are indicators of redox conditions of the hydrothermal solution including results of field measurements of redox conditions made using an oxidation-reduction potential (ORP) probe.

ORP, temperature and pH readings obtained in Summer 2009 for Bison Pool (four years after the biofilm sampling for metagenomic analysis and acquisition of chemical data reported in Table 2) and other flowing hot springs in Yellowstone are listed in Table 6 and in Dataset S5. The ORP measurements were obtained at three sites at Bison Pool that approximated the original locations of sites 3, 4 and 5. ORP, pH and temperature measurements at higher temperatures were also obtained at Mound Spring, which is the official name a nearby hot spring in Sentinel Meadows with chemical features similar to Bison Pool [55].

**Table 6:** Meter readings for selected hot springs and their outflows in Yellowstone National Park, Summer 2009.

Sample	Area	Spring	Latitude	Longitude	pH	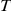 , °C	ORP, mV
090723G	Greater Obsidian Pool Area	1	0544526	4939786	5.12	79.0	27
090723K	Greater Obsidian Pool Area	1	0544541	4939799	5.38	57.6	98
090723F	Greater Obsidian Pool Area	2	0544482	4939773	4.21	68.3	185
090723E	Greater Obsidian Pool Area	2	0544497	4939806	4.21	53.0	183
090724PA	Sentinel Meadows	3	0511112	4934624	8.28	93.9	−258
090724OA	Sentinel Meadows	3	0511112	4934623	8.31	87.7	−227
090724NA	Sentinel Meadows	3	0511093	4934632	8.76	66.4	−98
090724O1	Sentinel Meadows	4	0510717	4935156	7.82	75.7	−55
090724P1	Sentinel Meadows	4	0510722	4935156	7.96	70.1	−58
090724Q1	Sentinel Meadows	4	0510723	4935156	8.06	66.2	−41
090724UA	Sentinel Meadows	5	0510846	4934731	7.84	87.3	−217
090724VA	Sentinel Meadows	5	0510842	4934733	8.07	71.3	−155
090728NA	Crater Hills	6	0541100	4944727	3.69	90.0	−50
090728-13	Crater Hills	6	0541100	4944727	3.50	51.6	274
090729DA	South of Sylvan Springs	7	0518409	4949162	5.84	79.5	−234
090729GA	South of Sylvan Springs	7	0518405	4949180	7.55	57.5	−130
090729RA	South of Sylvan Springs	7	0518395	4949185	7.92	44.9	−47
090729MA	South of Sylvan Springs	8	0518426	4949136	5.57	86.2	−248
090729HA	South of Sylvan Springs	8	0518426	4949144	6.17	73.3	−175
090729PA	South of Sylvan Springs	8	0518419	4949157	7.42	50.4	−42
090801HA	Heart Lake, Fissure Area	9	0538074	4905829	8.67	92.4	−373
090801I1	Heart Lake, Fissure Area	9	0538068	4905836	8.70	88.4	−351

**Figure 8:**
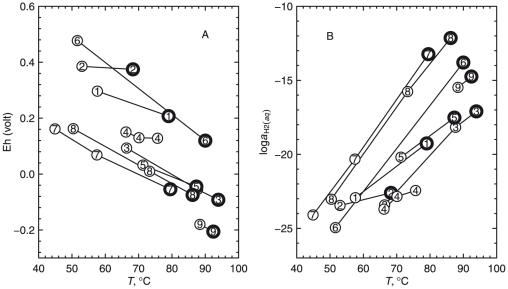
Calculated Eh and activity of hydrogen in hot springs. Eh and equilibrium 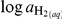 as functions of temperature for various hot springs were calculated from measured values of pH and ORP listed in Table 6. The different hot springs are identified by numbers (see Table 6 for key), and bold symbols indicate the sources of the hot springs.

The column labeled “spring” contains a unique number code for each hot spring and is used to label the points in Fig. 8. Named hot springs are Obsidian Pool (1), Mound Spring (3), Crater Hills Geyser (6). “Bison Pool” is number 4. Data for Bison Pool shown in this table were obtained in 2009 at three locations along the outflow channel. Latitude and longitude are the northing and easting, in meters, for the 12T grid zone for the Universal Transverse Mercator (UTM) projection using the WGS 84 datum. Supporting Dataset S5 includes the data in this table and estimated uncertainty in pH and ORP measurements.

Temperature and pH were measured in the field with hand-held temperature/conductivity (YSI, Yellow Springs, Ohio) and pH meters (WTW, Weilheim, Germany 300i pH meter with SenTix 41 pH electrode). Oxidation-reduction potential was measured using a high-temperature ORP probe (PI-M11-ORP-HT) rated to greater than 80°C and a Thermo Scientific pH/mV meter with a readout sensitivity of 1 mV, both acquired from Pulse Instruments (Van Nuys, CA). The ORP probe contains a silver-silver chloride (Ag/AgCl) reference electrode with saturated KCl solution. Before the field work, the ORP meter was calibrated in the laboratory at 25°C using a stock of Light's Solution (ferrous/ferric sulfate in sulfuric acid) supplied by Pulse Instruments. The stated potential of the solution is 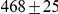 mV at 25°C vs. saturated KCl/AgCl electrode.

To convert the ORP readings (referenced to the Ag/AgCl electrode) to Eh (referenced to the standard hydrogen electrode, or SHE) we used
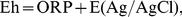
(27)where E(Ag/AgCl) is the potential vs. SHE of the Ag/AgCl reference electrode. The potential of the reference electrode was calculated using an equation for Ag/AgCl with 1M KCl electrolyte [57]

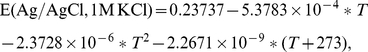
(28)where 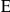 is in volts and 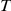 is in degrees Celsius. The effect of differences between the saturated and 1M KCl Ag/AgCl electrodes is discussed further below.

Values of Eh calculated by combining field measurements of ORP with Eqs. (27) and (28) are shown in Fig. 8a. The hot springs are identified by the number codes given in Table 6, and the source pools are indicated by bold symbols. The values for Mound Spring and Bison Pool (points labeled “3” and “4” in the figure) increase with decreasing temperature and are similar to an unnamed hot spring in Sentinel Meadows for which data are available (points labeled “5” in the figure; the GPS locations of this and other springs are given in Table 6).

Equilibrium values of 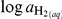 were calculated by combining Eh, pH and temperature. The values of Eh were first converted to pe (the negative of the logarithm of the activity of the electron) using [26]

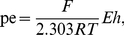
(29)where 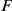 denotes the Faraday constant. Then, 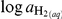 was calculated from the law of mass action for
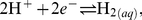
(30)that is,
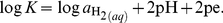
(31)Values of 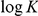 calculated using the standard molal properties of the species in Reaction 30 (see Methods) were combined with pe and pH to generate the values of 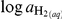 shown in Fig. 8b. These values correspond to equilibrium with both protons (activities constrained by pH measurements) and electrons (activities inferred from the ORP measurements, which does not capture the full spectrum of reactivity of electrons in the solution).

At 25°C the potential of the Ag/AgCl electrode (1M KCl) calculated using Eq. (28) is 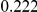 V. In contrast, the potential of the Ag/AgCl electrode with saturated KCl, which might be a more appropriate choice for calculations given the specifications of the ORP probe used for the measurements, is about 0.197V [58], or about 0.025V lower. Eqs. 29 and 31 can be used to calculate that a decrease of 0.025V at constant pH would increase the equilibrium 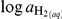 by approximately 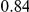 at 25°C and 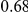 at 100°C. Changes of this magnitude would not drastically affect the relative positions of the points and lines shown in Fig. 8a or the comparisons drawn further below. The difference between the potentials of the 1M and saturated KCl electrode is probably also comparable in size to the total uncertainty in the measurement of oxidation-reduction potential in reactive hydrothermal fluids (see for example Ref. [59]). Therefore, the values of E(Ag/AgCl) used to calculate Eh from ORP (Eq. 27) were taken from Eq. (28) without modification.

Although the water at Mound Spring had a higher pH than reported for Bison Pool (see Table 6) the equilibrium values of 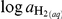 calculated using Reaction 30 for the two hot springs are close to each other at similar temperatures. The values of 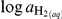 calculated in this manner for the outflow channels of Bison Pool and Mound Spring are lower than other hot springs at the same temperatures shown in Fig. 8b, but a more reduced fluid at higher temperatures seems to be the case for any of the measured hot springs.

### Comparison of equilibrium activities of hydrogen

Concentrations of chemical species such as dissolved oxygen, and species with an element in different oxidation states (e.g. sulfide and sulfate) can be expressed on the 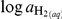 redox scale using relationships derived from chemical equilibrium. Values of 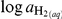 for equilibrium between dissolved sulfide (as 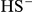) and sulfate (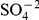) were obtained using the law of mass action for
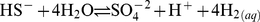
(32)at the temperature of each sampling site. The law of mass action is the relationship (Eq. 4) between the equilibrium constant (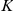) and activity product (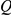) of this reaction when the chemical affinity (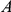) of the reaction is zero (see Eq. 31 for an example). Activities of 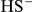 and 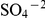 were taken to be equal to molalities of the species listed in Table 2, and values of 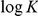 were again obtained using standard molal thermodynamic properties of the species as a function of temperature calculated as described in the Methods.

Values of 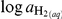 in equilibrium with dissolved oxygen were obtained using the law of mass action for
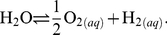
(33)using activities of 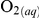 derived from the concentrations listed in Table 2.

**Figure 9:**
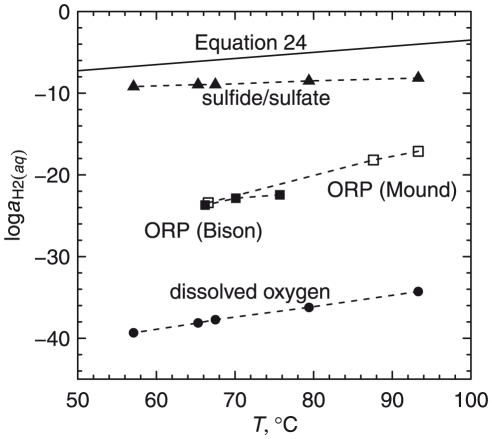
Comparison of different estimates of hydrogen activity as a function of temperature. Values shown for sulfide/sulfate and dissolved oxygen were computed using measured concentrations of those species listed in Table 2. Values shown for “Eh” were computed using pHs and ORP readings listed in Table 6. The solid line indicates values calculated using Eq. (24).

Fig. 9 shows a comparison of the activities of hydrogen calculated using Eq. (24) and activities of hydrogen in equilibrium with measured oxidation-reduction potentials (ORP) and the oxygen and sulfur redox indicators described above. The temperature dependence of the equilibrium 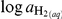 is strongest for the ORP measurements and weakest for the sulfur system. Among the three different redox indicators considered here (ORP, sulfide/sulfate, and dissolved oxygen) the lowest values of 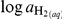 come from equilibrium with dissolved oxygen and the highest for equilibrium in the sulfide/sulfate reaction. All of the redox indicators have lower equilibrium values of 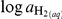 than those calculated using Eq. (24).

These three proxies are limiting cases, indicating what the values of 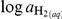 would be if each of the reactions dictated the actual hydrogen activity in the system. Therefore, the separation of the lines in Fig. 9 provides evidence that overall redox equilibrium does not characterize the hot spring (see Ref. [55] for further evidence of redox disequilibria in hot springs). Nevertheless, the simplest explanation for the trends shown in Fig. 9 is that the states of various redox reactions in the water are all becoming more oxidized with decreasing temperature, and that the redox gradient derived from the relative stabilities of the proteins (Eq. 24) occurs at more reducing conditions than any of the inorganic oxidation-reduction reactions.

## Discussion

By computing the standard Gibbs energies of overall reactions representing the formation of proteins, and using geochemical data on the gradients of temperature and chemistry, the relative stabilities of model proteins derived from metagenomic sequences in a hot spring could be calculated. It was possible to compute relative stabilities of the model proteins that reflect their overall spatial distribution in the system. An equation for hydrogen activity as a function of temperature was proposed, as a way of calibrating the model to maximize the correspondence between the geographical distributions of the sampling sites and the progression of relative stabilities of proteins.

There is a general increase in average oxidation state of carbon in the model proteins that parallels the rising redox potential of species in the water as it flows away, also cooling, from the hot spring. However, the average oxidation states of carbon in the different classes of model proteins are offset from each other, with membrane-associated proteins (including permeases, transport proteins and oxidoreductases) being more reduced. As noted above, hydrophobic amino acids tend to have lower oxidation states of carbon, so an increase in the number of hydrophobic amino acids can account for a decrease in average oxidation state of carbon in the proteins. An increased frequency of hydrophobic amino acids in proteins is a likely feature of adaptation to higher temperatures, as seen in the genomes of model organisms [60], [61]. Our metagenomically based finding of a negative correlation between temperature and oxidation state of carbon is consistent with those results.

The oxidation states of carbon in the different classes of model proteins increase along the outflow channel of the hot spring, except for a slight decrease between sites 4 and 5. The apparent inversion in relative stabilities between sites 4 and 5 might be connected to additional hydrogen input, possibly from oxygenic photosynthesizers [62], as a byproduct of nitrogen fixation [63] or fermentation, and/or from secondary sources of reduced gases in the hot-spring system.

A distinctive feature of the chemical compositions of the proteins in the study area is the departure of site 3 from the general trends for H/C, O/C and S/C shown in Fig. 3, the infrequent appearance of the model proteins for site 3 in the diagrams in Fig. 6, and the generally low equilibrium degrees of formation of these proteins in Fig. 7. Therefore, the model proteins for site 3 are on average less stable, or have a greater energy of formation, than those from other sites. This result could imply that there are specific metabolic requirements of the organisms at site 3, near the photosynthetic fringe, that cause them to produce proteins that are relatively energetically demanding.

The calculations described here may help elucidate the redox gradients between and cellular interiors and their surroundings. The interiors of bacterial cells such as *Escherichia coli* are more reduced than the growth medium in laboratory experiments [64], but much remains unknown about the redox conditions of microbial interiors in high-temperature environments. Fig. 9 shows that effective values of 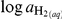 in equilibrium with sulfur/sulfide, dissolved oxygen, and inferred Eh values, all measured on bulk water samples in the field, decrease as as a function of temperature (oxygen and Eh more so), but that the protein-equilibrium-based model equation for 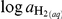 is more reduced than any of these proxies. This finding implies that the interiors of the microbial cells, where the proteins are mainly present, are more reduced than the environmental conditions at the temperatures in the hot spring. There is, however, not a general agreement about the redox conditions inside microbial cells; relatively oxidized conditions would help to account for the probable frequent occurrence of disulfide bonds in proteins in archaeal organisms (including some hyperthermophilic representatives) [65]. It would be useful to have data on subcellular redox indicators (e.g. oxidized and reduced forms of glutathione) at high temperatures to help resolve these questions.

Outlining the convergence of physical, chemical and biological forces that shape the information present in metagenomic sequences would benefit from the development of more sophisticated thermodynamic models and a tighter connection with phylogenetic approaches. For example, the construction of the model proteins could be based on identification of housekeeping genes that are conserved across phyla [66]. The use of only aligned sequences therein would help to eliminate some of the noise inherent in averaging shotgun sequence fragments, leading to a closer resolution of the differences between proteins from different environments.

Comparing values such as the average oxidation state of carbon with the phylogenetic relationships of gene families that have appeared in different redox conditions [67] might reveal correlations between chemical composition of proteins and evolutionary constraints. Incorporating the energetics of protein-forming chemical reactions in such an analysis would permit even greater integration of available data on the organisms' environments. Comparative calculations of the energetics of overall protein formation reactions is conducive to integrative studies of microbial communities and environments because the energies depend on both molecular sequences and properties of the chemical environment. Extension of such an integrated thermodynamic framework could be a new way forward to quantifying the relationships between chemically distinct environments and their microbial communities.

## Supporting Information

Dataset S1 Script for the CHNOSZ package (version 0.9–5) for the R software environment [42] demonstrating the relative stability calculation for the example described in the Methods.
(TXT)

Dataset S2 Script for the CHNOSZ package including code to produce all of the figures (except the map) and Table 4. The code also depends on the files in Dataset S4 (for Fig. 3), Dataset S3 (for Figs. 4, 5, 6, 7) and Dataset S5 (for Fig. 8). Instructions for running the code are provided in the comments at the top of the file.
(TXT)

Dataset S3 Amino acid compositions of model proteins. This file was produced using the “mkprot” function contained in the source code listing of Dataset S2.
(TXT)

Dataset S4 Results of bootstrap analysis of the elemental ratios and average oxidation number of carbon for all protein sequences at each site. This file was produced using the “boot.prep” function contained in the souce code listing of Dataset S2.
(TXT)

Dataset S5 Temperature, pH and oxidation-reduction potential measurements in hot springs and outflow channels, Summer 2009. Northing and easting (in meters) are listed for the Universal Transverse Mercator projection using the 12T grid zone; elevation is in meters.
(TXT)

## References

[1] Acquisti C, Kleffe J, Collins S (2007). Oxygen content of transmembrane proteins over macroevolutionary time scales.. Nature.

[2] Nakashima H, Fukuchi S, Nishikawa K (2003). Compositional changes in RNA, DNA and proteins for bacterial adaptation to higher and lower temperatures.. J Biochem.

[3] Zeldovich KB, Berezovsky IN, Shakhnovich EI (2007). Protein and DNA sequence determinants of thermophilic adaptation.. PLoS Comput Biol.

[4] Boyd ES, Hamilton TL, Spear JR, Lavin M, Peters JW (2010). [FeFe]-hydrogenase in Yellowstone National Park: evidence for dispersal limitation and phylogenetic niche conservatism.. ISME J.

[5] Inskeep WP, Rusch DB, Jay ZJ, Herrgard MJ, Kozubal MA (2010). Metagenomes from hightemperature chemotrophic systems reveal geochemical controls on microbial community structure and function.. PLoS ONE.

[6] Akashi H, Gojobori T (2002). Metabolic efficiency and amino acid composition in the proteomes of *Escherichia coli* and *Bacillus subtilis*.. Proc Natl Acad Sci U S A.

[7] Barton MD, Delneri D, Oliver SG, Rattray M, Bergman CM (2010). Evolutionary systems biology of amino acid biosynthetic cost in yeast.. PLoS ONE.

[8] Wagner A (2005). Energy constraints on the evolution of gene expression.. Mol Biol Evol.

[9] Amend JP, Shock EL (1998). Energetics of amino acid synthesis in hydrothermal ecosystems.. Science.

[10] Amend JP, Helgeson HC (2000). Calculation of the standard molal thermodynamic properties of aqueous biomolecules at elevated temperatures and pressures. II. Unfolded proteins.. Biophys Chem.

[11] Dick JM, LaRowe DE, Helgeson HC (2006). Temperature, pressure, and electrochemical constraints on protein speciation: Group additivity calculation of the standard molal thermodynamic properties of ionized unfolded proteins.. Biogeosciences.

[12] Havig JR, Raymond J, Meyer-Dombard D, Zolotova N, Shock E (2011). Merging isotopes and community genomics in a siliceous sinter-depositing hot spring.. J Geophys Res - Biogeosciences.

[13] Meyer-Dombard DR, Swingley W, Raymond J, Havig J, Shock EL (2011). Hydrothermal ecotones and streamer biofilm communities in the Lower Geyser Basin, Yellowstone National Park.. Environ Microbiol.

[14] Buvet R, Milazzo G, Blank M (1983). General criteria for the fulfillment of redox reactions.. Bioelectrochemistry I: Biological Redox Reactions.

[15] Kroll JH, Donahue NM, Jimenez JL, Kessler SH, Canagaratna MR (2011). Carbon oxidation state as a metric for describing the chemistry of atmospheric organic aerosol.. Nature Chemistry.

[16] Masiello CA, Gallagher ME, Randerson JT, Deco RM, Chadwick OA (2008). Evaluating two experimental approaches for measuring ecosystem carbon oxidation state and oxidative ratio.. J Geophy Res - Biogeosciences.

[17] LaRowe DE, Van Cappellen P (2011). Degradation of natural organic matter: A thermodynamic analysis.. Geochim Cosmochim Acta.

[18] Hendrickson JB, Cram DJ, Hammond GS (1970). Organic Chemistry.

[19] International Union of Pure and Applied Chemistry IUPAC Compendium of Chemical Terminology -The Gold Book.. http://goldbook.iupac.org/.

[20] Calvert JG (1990). Glossary of atmospheric chemistry terms - (recommendations 1990).. Pure Appl Chem.

[21] Kyte J, Doolittle RF (1982). A simple method for displaying the hydropathic character of a protein.. J Mol Biol.

[22] Bowers TS, Jackson KJ, Helgeson HC (1984). Equilibrium Activity Diagrams for Coexisting Minerals and Aqueous Solutions at Pressures and Temperatures to 5 kb and 600°C.

[23] De Donder Th (1927). L'Affinité.

[24] Kondepudi DK, Prigogine I (1998). Modern Thermodynamics: From Heat Engines to Dissipative Structures.

[25] Helgeson HC, Kirkham DH, Flowers GC (1981). Theoretical prediction of the thermodynamic behavior of aqueous electrolytes at high pressures and temperatures. IV. Calculation of activity coefficients, osmotic coefficients, and apparent molal and standard and relative partial molal properties to 600°C and 5 Kb.. Am J Sci.

[26] Drever JI (1997). The Geochemistry of Natural Waters.

[27] Dick JM (2008). Calculation of the relative metastabilities of proteins using the CHNOSZ software package.. Geochem Trans.

[28] Haney PJ, Badger JH, Buldak GL, Reich CI, Woese CR (1999). Thermal adaptation analyzed by comparison of protein sequences from mesophilic and extremely thermophilic *Methanococcus* species.. Proc Natl Acad Sci U S A.

[29] Akca E, Claus H, Schultz N, Karbach G, Schlott B (2002). Genes and derived amino acid sequences of S-layer proteins from mesophilic, thermophilic, and extremely thermophilic methanococci.. Extremophiles.

[30] Shock E, Canovas P (2010). The potential for abiotic organic synthesis and biosynthesis at seafloor hydrothermal systems.. Geofluids.

[31] Spear JR, Walker JJ, McCollom TM, Pace NR (2005). Hydrogen and bioenergetics in the Yellowstone geothermal ecosystem.. Proc Natl Acad Sci U S A.

[32] Hoehler TM, Alperin MJ, Albert DB, Martens CS (1998). Thermodynamic control on hydrogen concentrations in anoxic sediments.. Geochim Cosmochim Acta.

[33] Tanger JC, Helgeson HC (1988). Calculation of the thermodynamic and transport properties of aqueous species at high pressures and temperatures: Revised equations of state for the standard partial molal properties of ions and electrolytes.. Am J Sci.

[34] Shock EL, Helgeson HC, Sverjensky DA (1989). Calculation of the thermodynamic and transport properties of aqueous species at high pressures and temperatures: Standard partial molal properties of inorganic neutral species.. Geochim Cosmochim Acta.

[35] Shock EL, Oelkers EH, Johnson JW, Sverjensky DA, Helgeson HC (1992). Calculation of the thermodynamic properties of aqueous species at high pressures and temperatures: Effective electrostatic radii, dissociation constants, and standard partial molal properties to 1000°C and 5 kbar.. J Chem Soc, Faraday Trans.

[36] Levelt-Sengers JMH, Kamgarparsi B, Balfour FW, Sengers JV (1983). Thermodynamic properties of steam in the critical region.. J Phys Chem Ref Data.

[37] Haar L, Gallagher JS, Kell GS (1984). NBS/NRC Steam Tables.

[38] Johnson JW, Norton D (1991). Critical phenomena in hydrothermal systems: state, thermodynamic, electrostatic, and transport properties of H2O in the critical region.. Am J Sci.

[39] Johnson JW, Oelkers EH, Helgeson HC (1992). SUPCRT92: A software package for calculating the standard molal thermodynamic properties of minerals, gases, aqueous species, and reactions from 1 to 5000 bar and 0 to 1000°C.. Comp Geosci.

[40] Shock EL, Helgeson HC (1988). Calculation of the thermodynamic and transport properties of aqueous species at high pressures and temperatures: Correlation algorithms for ionic species and equation of state predictions to 5 kb and 1000°C.. Geochim Cosmochim Acta.

[41] Schulte MD, Shock EL, Wood RH (2001). The temperature dependence of the standard-state thermodynamic properties of aqueous nonelectrolytes.. Geochim Cosmochim Acta.

[42] R Development Core Team (2011). R: A Language and Environment for Statistical Computing.. http://www.R-project.org.

[43] Zimmerman SB, Minton AP (1993). Macromolecular crowding: Biochemical, biophysical, and physiological consequences.. Annu Rev Biophys Biomolec Struct.

[44] Curtis RA, Prausnitz JM, Blanch HW (1998). Protein-protein and protein-salt interactions in aqueous protein solutions containing concentrated electrolytes.. Biotechnol Bioeng.

[45] Engel T, Reid P (2006). Thermodynamics, Statistical Thermodynamics, and Kinetics.

[46] Nelson PG (1986). Treatment of chemical equilibrium without using thermodynamics or statistical mechanics.. J Chem Ed.

[47] Pfeil W (1998). Protein Stability and Folding.

[48] Privalov PL, Khechinashvili NN (1974). A thermodynamic approach to the problem of stabilization of globular protein structure: A calorimetric study.. J Mol Biol.

[49] Meyer-Dombard DR, Shock EL, Amend JP (2005). Archaeal and bacterial communities in geochemically diverse hot springs of Yellowstone National Park, USA.. Geobiology.

[50] Huber R, Eder W, Heldwein S, Wanner G, Huber H (1998). *Thermocrinis ruber* gen. nov., sp. nov., a pink-filament-forming hyperthermophilic bacterium isolated from Yellowstone National Park.. Appl Environ Microbiol.

[51] Ruff-Roberts AL, Kuenen JG, Ward DM (1994). Distribution of cultivated and uncultivated cyanobacteria and *Chloroflexus*-like bacteria in hot spring microbial mats.. Appl Environ Microbiol.

[52] Cox A, Shock EL, Havig JR (2011). The transition to microbial photosynthesis in hot spring ecosystems.. Chem Geol.

[53] Markowitz VM, Ivanova NN, Szeto E, Palaniappan K, Chu K (2008). IMG/M: a data management and analysis system for metagenomes.. Nucleic Acids Res.

[54] McInerney MJ, Rohlin L, Mouttaki H, Kim U, Krupp RS (2007). The genome of *Syntrophus aciditrophicus*: Life at the thermodynamic limit of microbial growth.. Proc Natl Acad Sci U S A.

[55] Shock E, Holland M, Meyer-Dombard D, Amend J, Osburn G (2010). Quantifying inorganic sources of geochemical energy in hydrothermal ecosystems, Yellowstone National Park, U.S.A.. Geochim Cosmochim Acta.

[56] McCollom TM, Amend JP (2005). A thermodynamic assessment of energy requirements for biomass synthesis by chemolithoautotrophic micro-organisms in oxic and anoxic environments.. Geobiology.

[57] Bard AJ, Parsons R, Jordan J (1985). Standard Potentials in Aqueous Solution.

[58] Sawyer D, Roberts JL (1974). Experimental Electrochemistry for Chemists.

[59] Oijerholm J, Forsberg S, Hermansson HP, Ullberg M (2009). Relation between the SHE and the internal Ag/AgCl reference electrode at high temperatures.. J Electrochem Soc.

[60] De Vendittis E, Castellano I, Cotugno R, Ruocco MR, Raimo G (2008). Adaptation of model proteins from cold to hot environments involves continuous and small adjustments of average parameters related to amino acid composition.. J Theor Biol.

[61] McDonald JH (2001). Patterns of temperature adaptation in proteins from the bacteria *Deinococcus radiodurans* and *Thermus thermophilus*.. Mol Biol Evol.

[62] Hoehler TM, Bebout BM, Des Marais DJ (2001). The role of microbial mats in the production of reduced gases on the early Earth.. Nature.

[63] Lynch JM, Poole NJ (1979). Microbial Ecology: A Conceptual Approach.

[64] Hwang C, Sinskey AJ, Lodish HF (1992). Oxidized redox state of glutathione in the endoplasmic reticulum.. Science.

[65] Mallick P, Boutz DR, Eisenberg D, Yeates TO (2002). Genomic evidence that the intracellular proteins of archaeal microbes contain disulfide bonds.. Proc Natl Acad Sci U S A.

[66] Wu M, Eisen JA (2008). A simple, fast, and accurate method of phylogenomic inference.. Genome Biol.

[67] Duval S, Ducluzeau AL, Nitschke W, Schoepp-Cothenet B (2008). Enzyme phylogenies as markers for the oxidation state of the environment: The case of respiratory arsenate reductase and related enzymes.. BMC Evol Biol.

